# Enhancing the Biological Properties of Organic–Inorganic Hybrid Calcium Silicate Cements: An In Vitro Study

**DOI:** 10.3390/jfb15110337

**Published:** 2024-11-10

**Authors:** Minji Choi, Jiyoung Kwon, Ji-Hyun Jang, Duck-Su Kim, Hyun-Jung Kim

**Affiliations:** 1 Medical Science Research Institute, Kyung Hee University Medical Center, Seoul 02447, Republic of Korea; cmj8466@gmail.com; 2 Department of Conservative Dentistry, Kyung Hee University Dental Hospital, Seoul 02447, Republic of Korea; jykt55@naver.com; 3 Department of Conservative Dentistry, School of Dentistry, Kyung Hee University, Seoul 02447, Republic of Korea; jangjihyun@khu.ac.kr (J.-H.J.); dentist96@khu.ac.kr (D.-S.K.)

**Keywords:** hydraulic calcium silicate cement, elastin-like polypeptide, bioactive glass, cell viability, cell migration, osteogenesis, human periodontal ligament fibroblasts

## Abstract

(1) Background: This study aimed to enhance the biological properties of hydraulic calcium silicate cements (HCSCs) by incorporating organic and inorganic components, specifically elastin-like polypeptides (ELPs) and bioactive glass (BAG). We focused on the effects of these composites on the viability, migration, and osteogenic differentiation of human periodontal ligament fibroblasts (hPDLFs). (2) Methods: Proroot MTA was supplemented with 1–5 wt% 63S BAG and 10 wt% ELP. The experimental groups contained various combinations of HSCS with ELP and BAG. Cell viability was assessed using an MTT assay, cell migration was evaluated using wound healing and transwell assays, and osteogenic activity was determined through Alizarin Red S staining and a gene expression analysis of osteogenic markers (*ALP*, *RUNX-2*, *OCN*, and *Col1A2*). (3) Results: The combination of ELP and BAG significantly enhanced the viability of hPDLFs with an optimal BAG concentration of 1–4%. Cell migration assays demonstrated faster migration rates in groups with 2–4% BAG and ELP incorporation. Osteogenic activity was the highest with 2–3% BAG incorporation with ELP, as evidenced by intense Alizarin Red S staining and the upregulation of osteogenic differentiation markers. (4) Conclusions: The incorporation of ELP (organic) and BAG (inorganic) into HCSC significantly enhances the viability, migration, and osteogenic differentiation of hPDLFs. These findings suggest that composite HCSC might support healing in destructed bone lesions in endodontics.

## 1. Introduction

Hydraulic calcium silicate cements (HCSCs) are widely used in endodontics as a retrograde filling material and for repairing tooth perforations [1,2]. Recently, it has also been employed as an orthograde filling material in non-surgical root canal treatments [3]. In these clinical scenarios, HCSC, as a non-resorbable filling material, is placed in direct contact with the external periodontium, playing a crucial role in the regeneration of periodontal tissues and surrounding bone. Despite several advantages, including biocompatibility and excellent sealing capability [4], there is a potential to improve bioactive endodontic materials derived from HCSC. These advancements could lead to more versatile materials with a wide range of clinical applications aimed at enhancing bone tissue regeneration and improving the outcomes of treatments for periapical lesions. This study explores the synergy of incorporating organic and inorganic components into HCSC, aiming to create a composite material with enhanced biological properties. The two principal components chosen for this enhancement are elastin-like polypeptide (ELP) as an organic component and bioactive glass (BAG) as an inorganic component.

Organic–inorganic hybrid materials combine the unique advantages of organic polymers and inorganic substances to create composites with superior properties [5]. These materials generally have closely mixed organic and inorganic components, yielding improved mechanical strength, biocompatibility, and bioactivity. ELPs are a class of biopolymers that mimic the properties of elastin, a key extracellular matrix protein [6]. They possess several important attributes for clinical applications [7], including the ability to undergo reversible phase transitions and facilitate cell-signaling processes [7,8]. Previous research suggests that integrating ELPs into the HCSC matrix can improve the material’s biological interactions and mechanical properties [9]. BAGs have emerged as powerful agents in bone and dentin regeneration due to their ability to adhere to bone and stimulate osteogenesis by releasing soluble silica and calcium ions, which induce the formation of a hydroxycarbonate apatite layer similar to the mineral phase of bone [10,11,12].

Various cell types reside around the apical periodontium or perforated roots, such as cementoblasts, endothelial cells, nerve cells, fibroblasts, osteoblasts, and a few progenitors/stem cells. Among these, periodontal ligament fibroblasts (PDLFs) have been suggested to possess multipotent capabilities, meaning they have the potential to differentiate into various cell types involved in healing [13]. Under appropriate conditions, PDLFs can differentiate into osteoblasts and cementoblasts, highlighting their potential role in periodontal regeneration [13,14,15]. Hanan et al. suggested that it is more appropriate to study human PDLFs rather than osteoblasts when investigating cells that attach to root-end-filling materials [16]. Additionally, PDLFs have been chosen to evaluate the biocompatibility of sealers [17].

By combining ELP and BAG, the resulting organic–inorganic hybrid material leverages the strengths of both components. ELPs provide flexibility, biocompatibility, and the ability to promote cell signaling and adhesion [18], while BAG contributes to the mechanical strength and osteogenic potential of composites [19].

This study aimed to determine the optimal ratio of ELP to BAG in HCSC composites to achieve superior biological characteristics. This study examined the effects of these organically and inorganically enhanced HCSC composites on the cellular viability, migration, hard tissue deposition capacity, and osteogenic differentiation of human PDLFs. By optimizing the composition of HCSC with ELPs and BAGs, this study sought to develop a novel endodontic material that not only addresses the current limitations of HCSC but also enhances its regenerative potential, ultimately leading to improved clinical outcomes in endodontic and periodontal therapies.

## 2. Materials and Methods

### 2.1. Materials

Proroot MTA (Dentsply Sirona, Tulsa, USA) was used as the main core for the hybrid endodontic filling material. The major constituents of MTA powder were tricalcium silicate (Ca_3_SiO_5_), dicalcium silicate (Ca_2_SiO_4_), tricalcium aluminate (Ca_3_Al_2_O_6_), bismuth oxide (Bi_2_O_3_), and calcium sulfate (CaSO_4_). For inorganic enhancement, 63S BAG (Bonding Chemical, USA) was selected and supplemented to HCSC at concentrations of 1–5 wt%. Hybrid powders were homogenously mixed in the Teflon-coated bowl. For organic incorporation, V125E8, a type of ELP, was used at a 0.3 liquid-to-powder (L/P) ratio to hybrid powders. The L/P ratio of the experimental hybrid HCSC remained constant throughout this study.

V125E8 was synthesized as described in previous studies [8,9]. The designation V125E8 refers to 125 repetitions of the amino acid sequence (Val-Pro-Gly-Xaa-Gly), where Xaa is valine (V), with the addition of octaglutamic acid as a functional group at the C-terminus. The synthesis procedure involved gene editing via the annealing and ligation of synthetic oligonucleotides (IDT Inc., Coralville, IA, USA), followed by plasmid transformation into BLR (DE3) *Escherichia coli* (EMD Millipore, Gibbstown, NJ, USA). The transformed *E. coli* was cultured and purified using the inverse transition cycling method. V125E8 has a transition temperature between 31 °C and 33 °C [8]. V125E8 was supplemented in 10 wt% liquid form to create the experimental composite material. It was selected based on its proven ability to improve the mechanical and bonding properties of composite materials in previous studies [9,20]. Experimental groups are listed in Table 1.

### 2.2. Preparation of Experimental Specimens and Eluates

Each powder was mixed with deionized water (DW) in Groups 0BG, 2BG, and 5BG at a 0.3 L/P ratio. Each powder in Groups 0BG-L, 1BG-L, 2BG-L, 3BG-L, 4BG-L, and 5BG-L was mixed with 10 wt% ELP solution at a 0.3 L/P ratio. Upon the addition of liquid, the mixture attains a viscosity similar to that of a thick cream. Typically, the initial setting occurs within 10 min. The hardening process continues over the course of approximately 24 h, during which the material achieves its full mechanical strength. For the cell viability assay, Alizarin Red S staining, osteogenic differentiation, wound healing, and transwell assays, 30 specimens from each group were prepared in custom-made Teflon-coated plastic molds with a diameter of 3 mm and a height of 7 mm. These specimens were stored in 100% humidity chambers at 37 °C for 2 days. Subsequently, the specimens were removed from the molds and stored in simulated body fluid (SBF) for 14 days, with the solution being replaced every 2 days. The synthesis of SBF was based on the protocol described by Kokubo et al. [21]. The columnar specimens were sterilized by exposure to ultraviolet light for 30 min. Each specimen was then soaked for 5 days in 20 mL of stromal cell basal medium (SCBM) at 37 °C with 5% CO_2_ and 95% humidity. After 5 days, the media were collected, filtered using a sterilized Sartorius filter with a 0.22 μm pore membrane, and diluted in a 1:1 ratio in SCBM before testing on the cells. The collected eluates were utilized in experiments to evaluate how experimental groups influence cellular biological properties.

### 2.3. Cell Lines and Culture

Human PDLFs (hPDLFs) were purchased from Lonza (Basel, Switzerland). hPDLFs were grown in SCBM supplemented with a bullet kit (CC-3205; hFGF-B, insulin, hFGF-B, GA-1000, and 5% fetal bovine serum (FBS)) (Lonza, Basel, Switzerland) following the manufacturer’s instructions.

### 2.4. Cell Viability

Cell viability analysis of hPDLFs was performed after 1, 3, and 7 days using 3-(4,5-Dimethyl-2-thiazolyl)-2,5-diphenyl-2H-tetrazolium bromide (MTT) assay, as described previously [22]. Absorbance was measured (n = 3) at 570 nm using a microplate reader (Spark 10M, Tecan, Männedorf, Switzerland).

### 2.5. Cell Migration Assay

#### 2.5.1. Wound Healing Assay

An in vitro scratch wound healing model was used to assess cell migration. hPDLFs were seeded in 6-well plates at a density of 1 × 10^5^ cells per well. Twelve hours after seeding, the cells were treated with experimental media for 48 h. An artificial wound scratch was made in the cell monolayer, and the rate of wound closure was observed at 24 and 48 h and analyzed using ImageJ software (version 1.54).

#### 2.5.2. Transwell Assay

The in vitro invasive capacity of cells was measured using boyden chamber membranes (AP 48, Neuro Probe; Gaithersburg, MD, USA) coated with Matrigel for 5 h, and cells (2 × 10^4^ cells/well) were seeded onto the coated Matrigel. After 24 h of incubation, cells were visualized using 0.1% crystal violet [23].

### 2.6. Osteogenic Capability

#### 2.6.1. Alizarin Red S Staining Assay

hPDLFs were seeded in a 24-well cell culture plate at a density of 5 × 10^4^ cells/well. Cells were cultured in an osteogenic medium formulated with α-minimum essential medium supplemented with 10% FBS, gentamycin (50 μg/mL), dexamethasone (10 nM), L-ascorbic acid (100 μM), and β-glycerophosphate (10 mM) to promote osteogenic differentiation. For the negative control group, cells were maintained in a standard growth medium. The cells were incubated for a duration of two weeks, during which the respective media were freshly replaced every 2 to 3 days to ensure optimal cell growth and differentiation conditions. After 14 days of incubation, the media were removed, and the cells were thoroughly washed with phosphate-buffered saline to prepare for subsequent analyses. The staining protocol was slightly modified from that described by Jang et al. [24].

#### 2.6.2. Osteogenic Regulator Gene Expression Assay

Relative gene expression related to osteoblastic activity was determined using quantitative real-time polymerase chain reaction (qRT-PCR) [25]. To analyze gene expression following the induction of hPDLFs with osteogenic medium for 7 days, RNA was extracted using TRIzol^®^ reagent (Molecular Research Center Inc., Cincinnati, OH, USA) according to the manufacturer’s protocol. Total RNA (1 μg) isolated from cells was reverse-transcribed to cDNA using the PrimeScript™ 1st Strand cDNA Synthesis Kit (Takara Korea Biomedical Inc., Seoul, Republic of Korea). Amplification and monitoring of each cDNA were performed using qPCRBIO SyGreen Mix Hi-ROX (PCR Biosystems, London, UK) on a StepOnePlus™ Real-Time PCR system (Applied Biosystems; Thermo Fisher Scientific, Inc., Waltham, MA, USA). Specific primers used for RT-qCR are listed in Table 2. The qRT-PCR data were analyzed using the 2-ΔΔCT method, with *GAPDH* used as the housekeeping control. Fold changes in gene expression relative to the control group are shown as relative quantification values.

### 2.7. Statistical Analysis

The differences between the two groups were analyzed using a t-test to determine statistical significance. For multiple comparisons across groups, a one-way analysis of variance (ANOVA) was conducted, followed by Tukey’s post hoc test to identify specific group differences. Statistical analysis was performed using GraphPad Prism 10.1.1 (GraphPad Software Inc., San Diego, CA, USA).

## 3. Results

### 3.1. Cell Viability Test

The cell viability of hPDLFs was assessed using the MTT assay, with measurements taken on days 1, 3, and 7. A statistically significant increase was observed in cell viability over time for hPDLF (*p* < 0.0001). Specifically, cell viability was significantly higher on day 7 than on days 1 and 3 in all experimental groups (*p* < 0.0001).

**Figure 1:**
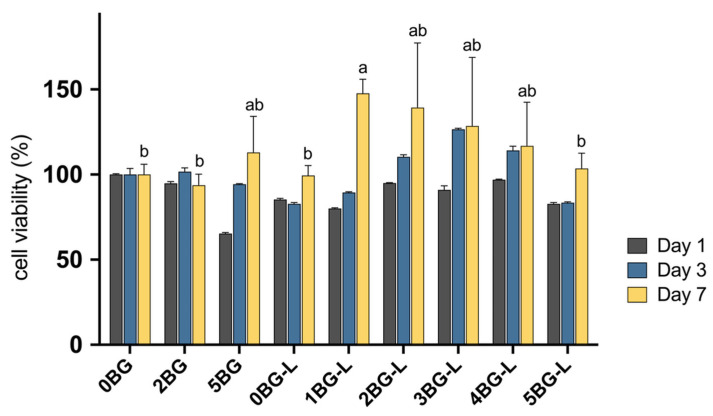
Cell viability in growth medium of experimental groups evaluated with MTT assay after 1, 3, and 7 days (n = 3). Same lowercase letters mean no statistical difference between groups of cell viability after 7 days.

The combination of BAG and ELP in HCSC significantly enhanced the viability of hPDLFs. The optimal BAG concentration was identified as 1–4%, which resulted in the highest cell viability at 7 days (*p* ≤ 0.0301) (Figure 1). No significant difference was observed in cell viability among the BAG concentrations of 1–4% (Groups 1, 2, 3, and 4BG-L) (*p* > 0.6326). However, in Group 5BG-L, a significant decrease was noted in viability compared with that in Group 1BG-L (*p* = 0.0301).

### 3.2. Cell Migration Assay

#### 3.2.1. Wound Healing Assay

**Figure 2:**
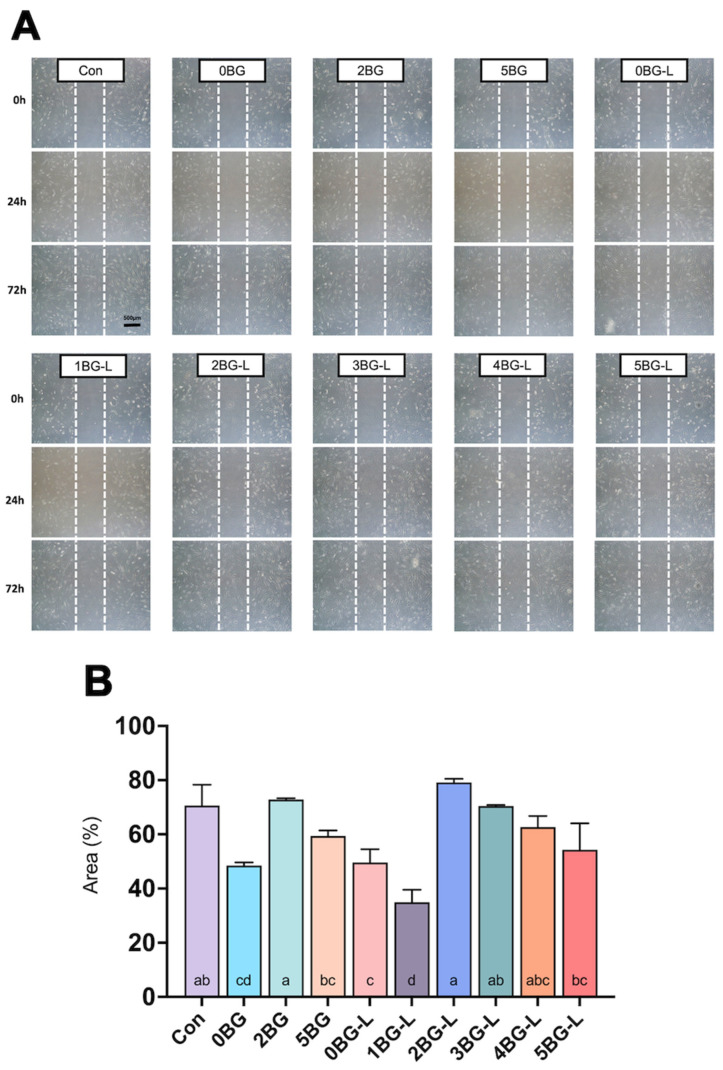
(**A**) Representative microscopic images of wound healing assay. (**B**) Relative cell-migrated area (%) of hPDLF in experimental groups (n = 3). Same lowercase letters inside bars mean no statistical difference between groups.

Figure 2A shows representative images of the relative area covered by hPDLFs that migrated into the scratched wound. Groups 2, 3, and 4BG-L exhibited similar cell migration to the negative control (*p* > 0.05), and Groups 2 and 3BG-L showed significantly higher migration ability than the positive control (Group 0BG) (*p* < 0.0192) (Figure 2B).

#### 3.2.2. Transwell Assay

**Figure 3:**
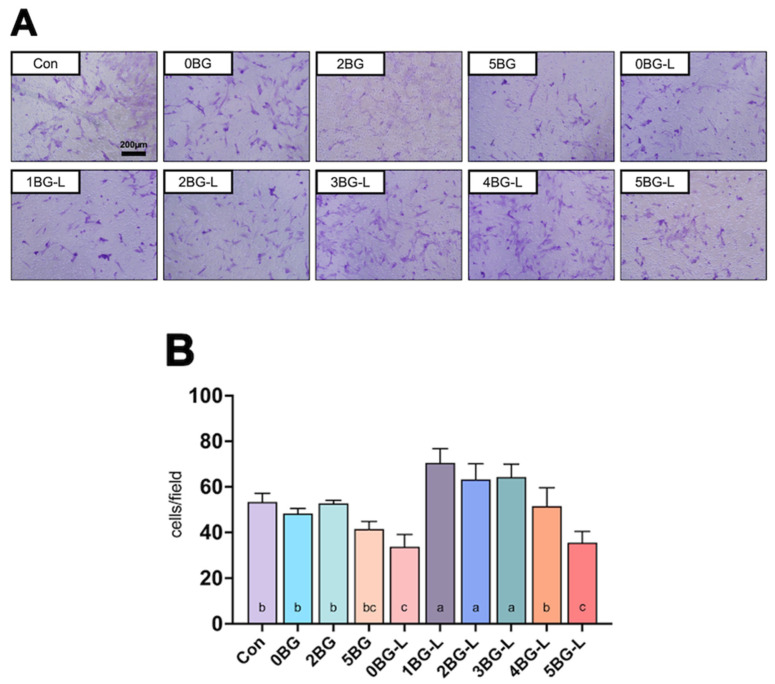
(**A**) Representative microscopic images of transwell assay. (**B**) Cell counts in microscopic images (n = 3) of experimental groups. Same lowercase letters mean no statistical difference between groups.

Figure 3A displays representative images of the transwell assay of hPDLFs. Groups 1, 2, and 3BG-L showed a significantly increased migration ability for hPDLFs compared to the positive control (*p* < 0.0108) and negative control groups (*p* < 0.0206) (Figure 3B).

### 3.3. Osteogenic Capability

#### 3.3.1. Alizarin Red S Staining Assay

**Figure 4:**
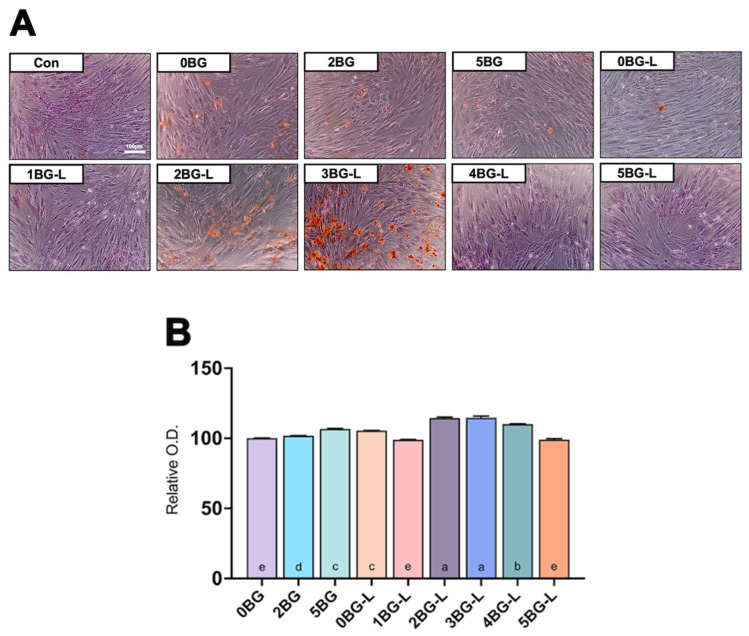
(**A**) Alizarin Red S staining assay of hPDLF. (**B**) Relative optical density (O.D.) for calcium detection (n = 3). Same lowercase letters mean no statistical difference between groups.

Figure 4A presents the results of the Alizarin Red S staining assay. Groups 2 and 3BG-L exhibited intense staining. Figure 4B shows a significant increase in absorbance at 405 nm, indicating elevated calcium deposits in Groups 2BG-L and 3BG-L (*p* < 0.0001).

#### 3.3.2. Osteogenic Regulator Gene Expression Assay

**Figure 5:**
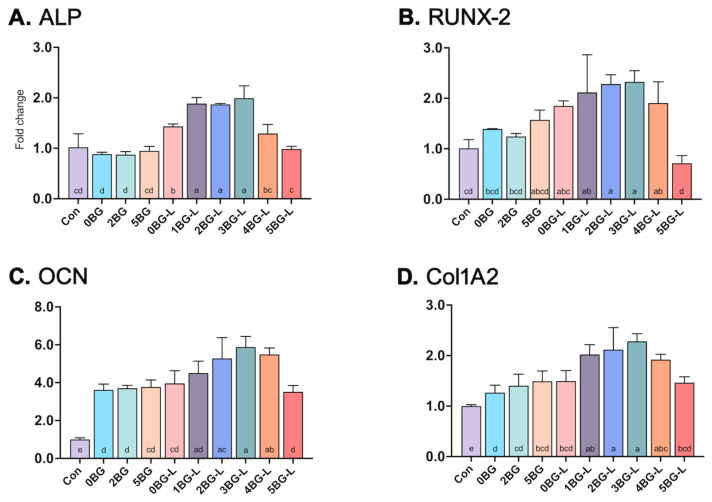
(**A**) Alkaline phosphatase (*ALP*), (**B**) runt-related transcription factor 2 (*RUNX-2*), (**C**) osteocalcin (*OCN*), and (**D**) Collagen Type I Alpha 2 Chain (*Col1A2*) gene expression of hPDLF in experimental groups (fold changes compared to negative control). Same lowercase letters mean no statistical difference between groups.

Four genes (*ALP*, *RUNX-2*, *OCN*, and *Col1A2*) were upregulated in the groups treated with the combinations of BAG and ELP (Figure 5). An increase of at least two-fold in the relative expression was observed for *ALP*, *RUNX-2*, and *Col1A2* in Groups 1, 2, and 3BG-L compared to that noted in the negative control (*p* < 0.0001) and positive control (Group 0BG; pure HCSC) (*p* < 0.0001). The *OCN* gene showed induction by almost 4–6-fold in Groups 1, 2, 3, and 4BG-L compared to the negative control (*p* < 0.0001). Groups 2, 3, and 4BG-L showed significant differences in the upregulation of the *OCN* gene compared with that noted with the positive control (Group 0BG) (*p* < 0.0286).

## 4. Discussion

This study examined improvement in the biological properties of HCSC by incorporating organic and inorganic components, specifically ELP and BAG. We evaluated the effects of the hybrid HSCSs on the viability, migration, and osteogenic differentiation of hPDLFs.

The MTT assay results demonstrate a significant increase in the cell viability of hPDLFs over time. The combination of BAG and ELP significantly enhanced the viability of hPDLFs at an optimal BAG concentration of 1–4%. The significant increase in cell viability observed in the experimental groups in which ELP was combined with BAG can be attributed to several factors based on the existing literature. ELPs have been shown to promote the proliferation and migration of fibroblasts, which are critical for wound healing and tissue regeneration [26]. Kinikoglu et al. reported that the inclusion of ELPs enhanced the self-renewal potential of oral epithelium and human lamina propria fibroblasts [27].

The wound healing assay results indicate a significant enhancement in cell adhesion and migration in the experimental groups wherein ELP was combined with BAG. This was particularly evident in Groups 2, 3, and 4BG-L, which exhibited a markedly faster migration of hPDLFs than the other groups. These findings align with the established role of ELP in promoting cell migration and adhesion, which are crucial components of the wound healing process [6,28]. Previous studies have demonstrated the efficacy of ELPs in accelerating fibroblast migration [29]. ELPs have been shown to facilitate the migration of mesenchymal stem cells and human fibroblasts by enhancing their motility and proliferation [30]. The presence of integrin- and laminin-binding domains within the ELP structure significantly contributes to this effect by improving cell–extracellular matrix interactions and promoting focal adhesion complex formation [30]. Especially since V125E8, a kind of ELP selected in this study, has hydroxyapatite-binding affinity [8], in the calcium- and silica-releasing environment in the presence of BAG, ELP can provide a conducive environment for cell attachment and spreading, which is crucial for cell migration. This increased cell adhesion is essential for the initial stages of wound healing, when cell migration into the wound site is necessary for tissue regeneration.

The Alizarin Red S staining assay showed intense staining in Groups 2BG-L and 3BG-L, indicating elevated calcium deposits. This suggests the enhanced osteogenic capabilities of these composites. The gene expression analysis further confirmed the upregulation of osteogenic markers, such as *ALP*, *RUNX-2*, *OCN*, and *Col1A2*, in the groups treated with the combinations of BAG and ELP. These results present the potential of these composites to promote bone regeneration and hard tissue formation.

The organic component selected to enhance our HCSC formulation was ELP, which possesses osteoconductive and osteoinductive properties [18]. ELP has been adopted as a major component of three-dimensional scaffolds for bone reconstruction [18,31]. The osteostimulatory properties of BAG are well documented and are primarily due to the release of multiple ions, such as calcium, phosphorus, and silicon ions, during its dissolution [18,32]. These ions, especially silicon ions, play a critical role in stimulating cellular activities essential for bone regeneration, including the proliferation and differentiation of osteoblasts [33].

Alkaline phosphatase (*ALP*) levels are an essential indicator of osteogenic differentiation. All inorganic and organically enhanced composites showed increased *ALP* activity compared to HCSC with only BAG, demonstrating the osteogenic differentiation of hPDLFs. In addition to BAG being known to increase *ALP* activity [34], ELP is also thought to contribute to the increase in *ALP*. Consequently, we propose that this composite formulation, especially in 2–4% BAG with ELP supplement, offers superior osteogenic potential compared to traditional HCSC, facilitating more efficient bone regeneration and improving clinical outcomes in endodontic applications, especially in destructed bone lesions.

This study has several limitations that should be addressed in future research. First, the investigation is limited to in vitro data. Future studies should include in vivo analyses to confirm the material’s suitability for endodontic applications. Second, although ELP and BAG are often used together as components of hybrid scaffolds [18,35], there is a lack of research on whether each component individually affects stem cell bioactivity or if they exert a combined chemical influence. Our study also does not elucidate the mechanisms through which these two materials enhance HCSC bioactivity. Further research is required to explore these mechanisms, which would offer deeper insights into the functionality of the experimental composite.

## 5. Conclusions

The findings of this study suggest that incorporating 10 wt% ELP (organic) solution and 2–3 wt% of BAG (inorganic) into HCSC can significantly enhance its biological properties in regard to the cell viability, migration, and osteogenic capacity of human PDLFs, making it a good bioactive hybrid material for endodontic applications. However, in vivo studies are necessary to validate these findings and explore the long-term effects of these composites in clinics.

## Figures and Tables

**Table 1:** Preparation of experimental groups. HCSC, hydraulic calcium silicate cement; DW, deionized water; MTA, mineral trioxide aggregates; %, wt%; ELP, 10 wt% elastin-like polypeptide solution.

	Groups	Liquid	Powder
Positive control	0BG	DW	100% MTA
Inorganic hybrid HCSC	2BG	DW	98% MTA + 2% BAG
	5BG	DW	95% MTA + 5% BAG
Organic–inorganic hybrid HCSC	0BG-L	ELP	100% MTA
	1BG-L	ELP	99% MTA + 1% BAG
	2BG-L	ELP	98% MTA + 2% BAG
	3BG-L	ELP	97% MTA + 3% BAG
	4BG-L	ELP	96% MTA + 4% BAG
	5BG-L	ELP	95% MTA + 5% BAG

**Table 2:** Forward and reverse primer sequences for reverse-transcription polymerase chain reaction.

Gene	Gene Name	Forward Primer Sequence	Reverse Primer Sequence
*ALP*	Alkaline phosphatase	5′-GACAAGAAGCCCTTCACTGC-3′	5′-AGACTGCGCCTGGTAGTTGT-3′
*RUNX-2*	Runt-related transcription factor 2	5′-GGTTAATCTCCGCAGGTCACT-3′	5′-CACTGTGCTGAAGAGGCTGTT-3′
*OCN*	Osteocalcin	5′-GGCGCTACCTGTATCAATGG-3′	5′-TCAGCCAACTCGTCACAGTC-3′
*Col1A2*	Collagen type I alpha 2	5′-CCTGGTGCTAAAGGAGAAAGAGG-3′	5′-ATCACCACGACTTCCAGCAGGA-3′
*hGAPDH*	Glyceraldehyde 3-phosphate dehydrogenase	5′-AACAGCGACACCCACTCCTC-3′	5′-CATACCAGGAAATGAGCTTGACAA-3′

## Data Availability

The original contributions presented in the study are included in the article, further inquiries can be directed to the corresponding author.

## References

[1] Lee S.-J., Monsef M., Torabinejad M. (1993). Sealing Ability of a Mineral Trioxide Aggregate for Repair of Lateral Root Perforations. J. Endod..

[2] Torabinejad M., Watson T.F., Pitt Ford T.R. (1993). Sealing Ability of a Mineral Trioxide Aggregate When Used as a Root End Filling Material. J. Endod..

[3] An H.J., Yoon H., Jung H.I., Shin D.-H., Song M. (2021). Comparison of Obturation Quality after MTA Orthograde Filling with Various Obturation Techniques. J. Clin. Med..

[4] Lim M., Jung C., Shin D.-H., Cho Y., Song M. (2020). Calcium Silicate-Based Root Canal Sealers: A Literature Review. Restor. Dent. Endod..

[5] Vallet-Regí M., Colilla M., González B. (2011). Medical Applications of Organic–Inorganic Hybrid Materials within the Field of Silica-Based Bioceramics. Chem. Soc. Rev..

[6] Sarangthem V., Singh T.D., Dinda A.K. (2021). Emerging Role of Elastin-Like Polypeptides in Regenerative Medicine. Adv. Wound Care.

[7] Despanie J., Dhandhukia J.P., Hamm-Alvarez S.F., MacKay J.A. (2016). Elastin-like Polypeptides: Therapeutic Applications for an Emerging Class of Nanomedicines. J. Control. Release.

[8] Wang E., Lee S.-H., Lee S.-W. (2011). Elastin-Like Polypeptide Based Hydroxyapatite Bionanocomposites. Biomacromolecules.

[9] Jang J.-H., Lee C.-O., Kim H.-J., Kim S.G., Lee S.-W., Kim S.-Y. (2018). Enhancing Effect of Elastinlike Polypeptide-Based Matrix on the Physical Properties of Mineral Trioxide Aggregate. J. Endod..

[10] Hench L.L., Jones J.R. (2015). Bioactive Glasses: Frontiers and Challenges. Front. Bioeng. Biotechnol..

[11] Baino F., Hamzehlou S., Kargozar S. (2018). Bioactive Glasses: Where Are We and Where Are We Going?. J. Funct. Biomater..

[12] Kim H.-J., Bae H.E., Lee J.-E., Park I.-S., Kim H.-G., Kwon J., Kim D.-S. (2021). Effects of Bioactive Glass Incorporation into Glass Ionomer Cement on Demineralized Dentin. Sci. Rep..

[13] Inanc B., Elcin A.E. (2006). Osteogenic Induction of Human Periodontal Ligament Fibroblasts Under Two- and Three-Dimensional Culture Conditions. Tissue Eng..

[14] Jönsson D., Nebel D., Bratthall G., Nilsson B.-O. (2011). The Human Periodontal Ligament Cell: A Fibroblast-like Cell Acting as an Immune Cell: Human Periodontal Ligament Cell Characteristics. J. Periodontal Res..

[15] Ivanovski S., Li H., Haase H.R., Bartold P.M. (2001). Expression of Bone Associated Macromolecules by Gingival and Periodontal Ligament Fibroblasts. J. Periodontal Res..

[16] Balto H., Al-Nazhan S. (2003). Attachment of Human Periodontal Ligament Fibroblasts to 3 Different Root-End Filling Materials: Scanning Electron Microscope Observation. Oral Surg. Oral Med. Oral Pathol. Oral Radiol. Endodontology.

[17] Szczurko G., Pawińska M., Łuczaj-Cepowicz E., Kierklo A., Marczuk-Kolada G., Hołownia A. (2018). Effect of Root Canal Sealers on Human Periodontal Ligament Fibroblast Viability: Ex Vivo Study. Odontology.

[18] Gurumurthy B., Tucci M.A., Fan L., Benghuzzi H.A., Pal P., Bidwell G.L., Salazar Marocho S.M., Cason Z., Gordy D., Janorkar A.V. (2020). Collagen-Elastin-Like Polypeptide-Bioglass Scaffolds for Guided Bone Regeneration. Adv. Healthc. Mater..

[19] Huang G., Xu L., Wu J., Wang S., Dong Y. (2021). Gelatin/Bioactive Glass Composite Scaffold for Promoting the Migration and Odontogenic Differentiation of Bone Marrow Mesenchymal Stem Cells. Polym. Test..

[20] Kim H.-J., Lee D., Cho S., Jang J.-H., Kim S.G., Kim S.-Y. (2019). Improvement of the Bonding Properties of Mineral Trioxide Aggregate by Elastin-Like Polypeptide Supplementation. Scanning.

[21] Kokubo T., Kushitani H., Sakka S., Kitsugi T., Yamamuro T. (1990). Solutions Able to Reproduce in Vivo Surface-structure Changes in Bioactive Glass-ceramic A-W. J. Biomed. Mater. Res..

[22] D’Antò V., Di Caprio M.P., Ametrano G., Simeone M., Rengo S., Spagnuolo G. (2010). Effect of Mineral Trioxide Aggregate on Mesenchymal Stem Cells. J. Endod..

[23] Justus C.R., Leffler N., Ruiz-Echevarria M., Yang L.V. (2014). In Vitro Cell Migration and Invasion Assays. J. Vis. Exp..

[24] Kwon S.-H., Jeong H.-J., Lee B.-N., Lee H.-S., Kim H.-J., Kim S.-Y., Kim D.-S., Jang J.-H. (2021). Effects of Various Mineral Trioxide Aggregates on Viability and Mineralization Potential of 3-Dimensional Cultured Dental Pulp Stem Cells. Appl. Sci..

[25] Frank O., Heim M., Jakob M., Barbero A., Schäfer D., Bendik I., Dick W., Heberer M., Martin I. (2002). Real-time Quantitative RT-PCR Analysis of Human Bone Marrow Stromal Cells during Osteogenic Differentiation in Vitro. J. Cell. Biochem..

[26] Kamoun A., Landeau J.-M., Godeau G., Wallach J., Duchesnay A., Pellat B., Hornebeck W. (1995). Growth Stimulation of Human Skin Fibroblasts by Elastin-Derived Peptides. Cell Adhes. Commun..

[27] Kinikoglu B., Rodríguez-Cabello J.C., Damour O., Hasirci V. (2011). The Influence of Elastin-like Recombinant Polymer on the Self-Renewing Potential of a 3D Tissue Equivalent Derived from Human Lamina Propria Fibroblasts and Oral Epithelial Cells. Biomaterials.

[28] Senior R.M., Griffin G.L., Mecham R.P. (1982). Chemotactic Responses of Fibroblasts to Tropoelastin and Elastin-Derived Peptides. J. Clin. Investig..

[29] Sarangthem V., Sharma H., Goel R., Ghose S., Park R.-W., Mohanty S., Chaudhuri T.K., Dinda A.K., Singh T.D. (2022). Application of Elastin-like Polypeptide (ELP) Containing Extra-Cellular Matrix (ECM) Binding Ligands in Regenerative Medicine. Int. J. Biol. Macromol..

[30] Sarangthem V., Sharma H., Mendiratta M., Sahoo R.K., Park R.-W., Kumar L., Singh T.D., Mohanty S. (2022). Application of Bio-Active Elastin-like Polypeptide on Regulation of Human Mesenchymal Stem Cell Behavior. Biomedicines.

[31] Gurumurthy B., Bierdeman P.C., Janorkar A.V. (2016). Composition of Elastin like Polypeptide–Collagen Composite Scaffold Influences in Vitro Osteogenic Activity of Human Adipose Derived Stem Cells. Dent. Mater..

[32] Borden M., Westerlund L.E., Lovric V., Walsh W. (2022). Controlling the Bone Regeneration Properties of Bioactive Glass: Effect of Particle Shape and Size. J. Biomed. Mater. Res. B Appl. Biomater..

[33] Damen J.J.M., Ten Cate J.M. (1992). Silica-Induced Precipitation of Calcium Phosphate in the Presence of Inhibitors of Hydroxyapatite Formation. J. Dent. Res..

[34] Reilly G.C., Radin S., Chen A.T., Ducheyne P. (2007). Differential Alkaline Phosphatase Responses of Rat and Human Bone Marrow Derived Mesenchymal Stem Cells to 45S5 Bioactive Glass. Biomaterials.

[35] Wheeler T.S., Sbravati N.D., Janorkar A.V. (2013). Mechanical & Cell Culture Properties of Elastin-Like Polypeptide, Collagen, Bioglass, and Carbon Nanosphere Composites. Ann. Biomed. Eng..

